# Use of Digital Diagnostic Aids for Initial Caries Detection: A Review

**DOI:** 10.3390/dj11100232

**Published:** 2023-09-28

**Authors:** Emma Kay Chan, Yuet Ying Wah, Walter Yu-Hang Lam, Chun-Hung Chu, Ollie Yiru Yu

**Affiliations:** Faculty of Dentistry, The University of Hong Kong, Hong Kong SAR, Chinaretlaw@hku.hk (W.Y.-H.L.); chchu@hku.hk (C.-H.C.)

**Keywords:** digital dentistry, dental caries, initial caries, caries detection, caries diagnosis

## Abstract

The advance in digital diagnostic technologies has significantly facilitated the detection of dental caries. Despite the increase in clinically available digital diagnostic aids for dental caries, there is yet to be a comprehensive summary of all available technology. This review aims to provide an overview of digital diagnostic aids for the clinical detection of dental caries, particularly those at an initial stage. Currently available digital diagnostic aids for caries detection can be classified into four categories according to the initial source of energy, including radiation-based aids, light-based aids, ultrasound-based aids, and electric-based aids. Radiation-based aids use ionizing radiation, normally X-ray, to produce images of dental structures. Radiation-based aids encompass digital bitewing radiography and cone beam computed tomography. Light-based aids employ light or laser to induce signals for the detection of the changes in the carious dental hard tissue. Common light-based aids include digital transillumination and light/laser-induced fluorescence. Ultrasound-based aids detect the signal of ultrasound waves to assess the acoustic impedance of the carious teeth. The ultrasound caries detector is an available ultrasound-based aid. Electric-based aids assess the changes in the electric current conductance or impedance of the teeth with caries. Available electric-based aids include electrical conductance measurement and alternating current impedance spectroscopy. Except for these clinically available digital diagnostic aids, many digital diagnostic aids for caries detection are still under development with promising results in laboratory settings.

## 1. Introduction

Dental caries, commonly known as tooth decay, is notably one of the most prevalent chronic diseases [1]. It is the gradual loss of tooth substance as a result of a complex interaction of the cariogenic bacteria in dental plaque biofilm and fermentable carbohydrates, leading to bacterial acid attack and disrupting the balance between the remineralization and demineralization of dental hard tissue [2,3]. A study from the Global Burden of Disease Collaborative Network estimated that 2.4 billion of the global population have untreated caries on permanent teeth, and 532 million children have untreated caries on primary teeth [4].

Undetected initial caries that later develop into deeper carious lesions might contribute to the high global prevalence of caries [5]. The current philosophy of caries management adopts a medical model focusing on caries prevention, nonrestorative management, and minimally invasive operative treatment [6]. The identification of dental caries, especially at an early stage, allows the preservation of healthy dental hard tissue with early intervention. Therefore, the detection and assessment of dental caries, especially at an early stage, are essential for the prevention and management of dental caries [5,7].

The most common and conventional method of detecting early caries is the visual-tactile technique [8]. This approach involves detecting the teeth’s visible colour change with naked eyes and the texture change of hard tissue lesions using a dental explorer [9]. Although this method is simple and cost-effective, a review has shown that it has low sensitivity and relatively high specificity for caries detection, making it unsuitable for early detection [10]. In addition, visual examination is not feasible for areas where direct visualization is inaccessible. A study also noted that using dental explorers during caries examinations can cause further damage to demineralized enamel structures and exacerbate the development of the carious lesion [11].

Due to the limitations of conventional caries detection approaches, the demand for novel caries detection approaches has emerged. Various technologies have been developed, particularly novel digital diagnostic aids that can identify lesions at an early stage. Digital aids are devices that utilize digital data for caries diagnosis and have become increasingly popular in various industries. Consequently, this review aims to provide an overview of digital diagnostic aids for the clinical detection of initial dental caries.

## 2. Literature Search

A comprehensive literature search was performed using keywords ((early caries) OR (initial caries)) AND ((digital) OR (light) OR (laser) OR (fluorescence) OR (transillumination)) AND ((Detection) OR (Assessment)) on the Pubmed, Scopus, and Web of Science databases. The keywords were chosen after an initial screening of articles related to digital diagnostic aids for caries. Several keywords including “light”, “laser”, “fluorescence”, and “transillumination” were found to be recurring in most articles. We have also included studies targeting early caries or initial lesions as this type of caries is the most difficult to detect and digital aids would be most useful in this case. Studies published on and before 31 December 2022 were selected and retrieved.

**Figure 1:**
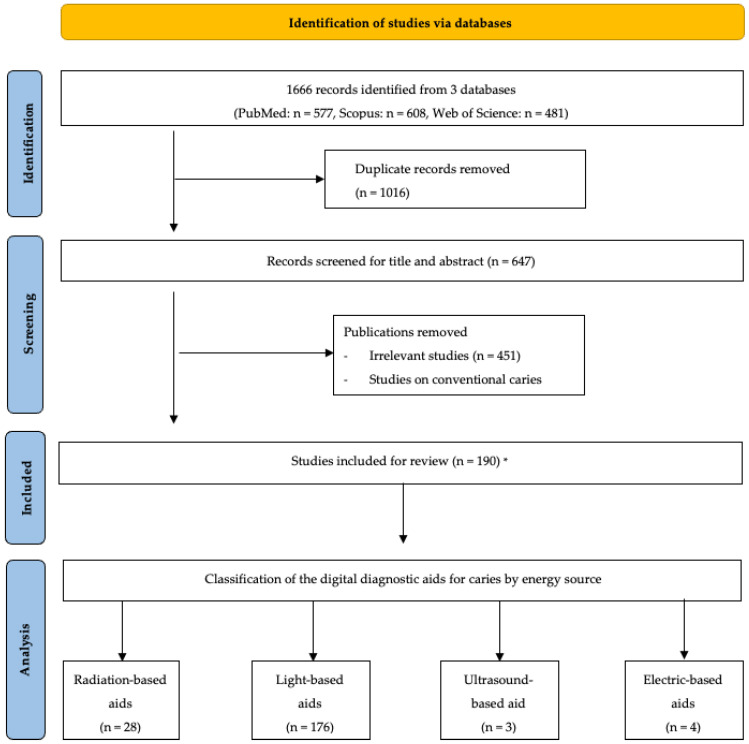
Flow chart of the study. * Among the 190 included studies, 18 of them investigated more than 1 type of diagnostic aid. 3 studies involved radiation-based, light-based, and electric-based aids. 14 studies involved radiation-based and light-based aids. 1 study involved light-based and electric-based aids.

A total of 1666 results were retrieved with 577 papers in Pubmed, 481 publications in Web of Science, and 608 publications in Scopus. After removing duplicates, 650 publications were included for further assessment. The titles and the abstracts of the studies were screened to include studies on digital diagnostic aids for the detection and assessment of dental caries (Figure 1). The articles deemed irrelevant were those that do not relate to human dental caries. 

**Figure 2:**
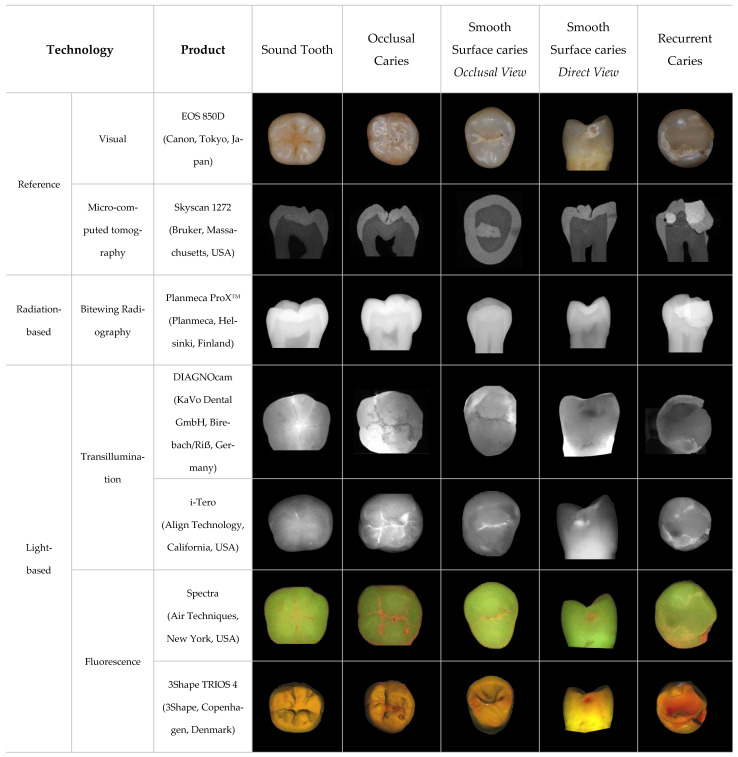
Images of carious teeth produced using digital caries diagnostic aids. Images of extracted human teeth that are sound, with occlusal caries, smooth surface caries, and recurrent caries were taken using various digital caries detection products, using those taken with a digital camera and micro-CT as references. These images are original, taken by the authors of this review.

The literature search identified 190 articles discussing various digital diagnostic aids that can be used for initial dental caries detection. Several digital diagnostic aids are clinically applicable with commercial products available (Table 1). These digital diagnostic aids can be categorized according to their energy sources. The primary energy sources that may be employed as digital signals to facilitate caries detection include X-rays, visible light, laser, coherence light, ultrasound, electricity, etc. Based on the energy source, these digital diagnostic aids for dental caries detection include radiation-based diagnostic aids, light-based diagnostic aids, ultrasound-based diagnostic aids, and electric-based diagnostic aids (Figure 2). Radiation-based diagnostic aids and light-based diagnostic aids provide images for caries detection. The representative images of radiation-based and light-based diagnostic aids for caries detection are thus shown in Figure 2. Ultrasound-based diagnostic aids and electric-based diagnostic aids commonly convert digital signals into numbers or spectrums as the result of caries detection.

Apart from these clinically available digital diagnostic aids, many digital aids for dental caries are still under development. These include optical coherent tomography, laser-induced thermal imaging, laser-induced acoustic spectroscopy, laser-induced breakdown spectroscopy, frequency-domain laser infrared photothermal radiometry, and modulated luminescence technology. Novel strategies that enhance the accuracy in the interpretation of the results of digital diagnostic aids are also under development. 

## 3. Digital Diagnostic Aids for Clinical Use

### 3.1. Radiation-Based Diagnostic Aids

X-ray, which is a form of electromagnetic radiation with specific wavelengths ranging from 10 nm to 0.01 pm, was introduced in the 1890s for the detection of dental caries [12]. Over time, digital radiography has gradually replaced conventional radiography due to its ease of image manipulation, enhanced image quality, and improved diagnostic accuracy [13]. A review found that digital radiography demonstrates higher accuracy compared with conventional analogue radiography [14]. Clinically used radiation-based diagnostic aids encompass digital bitewing radiographs and cone beam computed tomography.

#### 3.1.1. Digital Bitewing Radiographs

Digital bitewing radiography uses photographic film or digital detectors to capture images when X-rays interact with the emulsion on the film [12]. Digital bitewing radiographs are often employed right after a clinical examination of dental caries for approximal caries, occlusal caries, or secondary caries [15]. This technology provides a qualitative diagnosis by enabling the observation of lesion extension or density changes in enamel or dentine over time and allows for the long-term monitoring of carious lesions. [14,16]. However, certain limitations should be acknowledged. Firstly, the routine use of intraoral radiographs, especially for low-caries-risk patients, has been debated due to concerns about increased radiation risk [17]. Furthermore, such radiographs cannot distinguish between active and arrested carious lesions and sometimes between cavitated and non-cavitated surfaces, as the radiographic lesion depth does not always reflect the actual caries lesion [18]. Another consideration is that radiographs only detect lesions with a depth of at least 500 µm in the enamel. The inability to detect initial carious lesions limits its clinical value [19].

The accuracy of digital bitewing radiography varies largely, as reported by clinical studies on permanent teeth. Bitewing radiographs have a sensitivity of 0–0.93 [5,20,21] and a specificity of 0.6–1 [5,20,21] for the detection of occlusal caries. For the detection of approximal caries, the sensitivity and specificity values are 0.15–0.83 [5,22] and 0.6–0.99 [5,22], respectively. 

#### 3.1.2. Cone Beam-Computed Tomography

Dental cone beam-computed tomography (CBCT) utilizes a cone or pyramid-shaped X-ray beam with mostly flat panel detectors to construct three-dimensional high-resolution images that can be viewed in frontal, sagittal, and axial planes [23]. CBCT can be employed to detect caries in all sites of a tooth because it provides three-dimensional images. The main benefit of this technique is that it can overcome the limitations of two-dimensional imaging [24]. However, drawbacks such as radiation dose, costs, and imaging artefacts have been reported when using CBCT as a radiographic modality [17]. 

The accuracy of CBCT in detecting dental caries has shown considerable variability across different studies in the literature. Several in vitro studies showed that CBCT did not provide superior diagnostic accuracy in detecting enamel and dentine caries when compared with bitewing radiography [25,26,27]. Nonetheless, another study has revealed that CBCT exhibits a significantly higher sensitivity value when detecting cavitated approximal carious lesions than intraoral radiography [28]. For clinical studies in permanent dentition, CBCT has a reported sensitivity of 0.75–0.79 and a specificity of 0.77 in approximal lesions [28]. In general, CBCT is not advised as a primary diagnostic tool for routine caries detection.

### 3.2. Light-Based Diagnostic Aids

Light-based diagnostic aids employ various types of light or laser to generate signals and detect the changes of signals in carious teeth. Three major categories of light-based diagnostic aids are now available, including digital transillumination, light-induced fluorescence, and laser-induced fluorescence.

#### 3.2.1. Digital Transillumination

Transillumination is a technique of transmitting light through body tissues and assessing the density and composition of the tissue through the intensity of light [29]. Carious tissue with higher porosities absorbs more light and appears darker under transillumination [30]. Traditional fibre-optic transillumination cannot produce images and only allows the instant on-site interpretation of the examination result. To address this issue, digitalization has been incorporated into transillumination. The most common light source for digital transillumination is invisible near-infrared light oscillating between 700 nm and 1500 nm [31]. This light can penetrate deeper into dental tissues due to reduced scattering and absorption compared with visible white light in traditional transillumination, resulting in a better contrast between healthy and carious tissues [32]. The contrast in the light intensity in a tooth enables the clinician to observe the change in the light intensity in caries lesions. 

Digital transillumination is especially useful when detecting approximal caries [33]. Digital transillumination has the benefits of lower radiation dose, reduced patient discomfort, real-time image viewing, and overall higher feasibility than visual examination and radiography [33,34,35]. However, digital transillumination cannot accurately reveal a lesion’s size, volume, mineral content, or caries activity. Because it cannot distinguish carious lesions from developmental defects such as fluorosis, it may lead to overdiagnosis and overtreatment [36]. Moreover, the difficulties in capturing high-quality images may hinder caries detection [34].

Most of the studies on the accuracy of digital transillumination were performed on proximal caries. Digital transillumination showed sensitivity at 0.44–0.991 and specificity at 0.61–0.941 when detecting proximal caries in clinical studies [31,37]. 

#### 3.2.2. Light-Induced Fluorescence

Light with various wavelengths has been used to induce fluorescence in caries lesion, including ultraviolet light (100–400 nm), the green–yellow end of the visible light (370 nm), the blue–violet end of the visible light (400–450 nm), and near-infrared light (750–10,000 nm) [38,39,40]. Fluorescence is a type of luminescence, or the emission of light of longer wavelengths from a substance after absorbing light or other electromagnetic radiation at low wavelengths [41]. The use of fluorescence in caries detection is based on the phenomenon that carious lesions have altered fluorescence properties compared with sound dental tissues. When a carious lesion absorbs light of short wavelengths, it re-emits light at a longer wavelength, creating a colour change during the process [42]. 

Light-induced fluorescence can be applied to multiple surfaces of the carious tooth, including the occlusal surface, the buccal/lingual surface, or the proximal surface with reasonable interproximal space. It enables the quantification of the mineral loss in the caries lesion by capturing the average loss of fluorescence in carious tooth tissues compared with sound enamel and converting the loss of fluorescence into mineral density [43,44]. It also has the benefits of lower radiation dose, reduced patient discomfort, real-time image viewing, and storage, compared with visual examination and radiography. However, it cannot be applied to the area where light cannot access directly, such as a proximal surface of the tooth with an adjacent tooth presented. Moreover, because light might induce the generation of fluorescence from subjects other than carious teeth in the oral cavity, the interpretation of the detecting results should be careful to avoid overdiagnosis [45].

Clinical studies on the diagnostic accuracy of light-induced fluorescence are mostly based on devices that employed the blue–violet end of the visible light spectrum (400–450 nm). The accuracy of diagnostic aids adopting blue–violet light-induced fluorescence varies with the sites for detection. The sensitivity and specificity for occlusal caries were reported to be 0.26–0.92 and 0.41–0.1, respectively [5,21,46]. For the detection of approximal dental caries, the sensitivity and specificity values were 0.74 and 0.73, respectively [21]. For buccal caries, sensitivity and specificity were reported to be 0.74–0.85 and 0.49–0.80, respectively [5].

#### 3.2.3. Laser-Induced Fluorescence

Laser-induced fluorescence employs a red-light laser at a wavelength of greater than 655 nm to stimulate fluorescence in carious tissues [43]. Laser is an acronym for light amplification by stimulated emission of radiation. It is an electromagnetic wave generator that produces a single wavelength of light and focuses on a narrow beam [47,48]. The laser can induce fluorescence derived from protoporphyrin, a photosensitive pigment resulting from bacterial metabolic activities in carious lesions, to facilitate caries detection [49]. Because healthy dental tissues produce little or no fluorescence, the emitted fluorescence intensity correlates with the caries severity [49]. 

Laser-induced fluorescence can detect carious lesions from various sites of the tooth, including occlusal surfaces, smooth surfaces, and proximal surfaces. It can estimate the depth of the carious lesion and display a value from 0 to 99, in which lower scores indicate healthy tissues and higher scores reflect the need for restorative treatment [50,51]. It shares the same advantages as light-induced fluorescence, including a lower radiation dose, reduced patient discomfort, and real-time chairside detection compared with visual examination and radiography. Because the currently available laser-induced fluorescence devices display the results with numeric data, patient communication may be difficult compared with light-induced fluorescence devices. Moreover, laser-induced fluorescence has the risk of generating false positive results, which might lead to overdiagnosis [45].

Previous studies found that laser fluorescence presented a sensitivity value of 0.48–1 and a specificity value of 0.2–1 [5,46,52,53]. However, it is also prone to false-positive diagnoses, which may lead to overtreatment. Therefore, laser fluorescence is recommended to be used as a supplementary diagnostic aid instead of a primary diagnostic tool for caries detection. 

### 3.3. Electric-Based Diagnostic Aids

The use of electricity to detect caries was first proposed by Emile Magitot in 1878 [54]. Electric-based caries detection devices work based on the phenomenon that hydroxyapatite, which is the main component of dental enamel, has high electrical resistivity. When caries occur, the porosities in dental hard tissue increase in size and contain more electrically conductive fluids from the oral cavity compared with the sound tooth. Since the porosities are filled with this ionic fluid, there is a decrease in electrical resistance and an increase in electrical conductance [55,56]. Electric-based caries-detecting approaches can be categorized as electrical conductance measurement and alternating current impedance spectroscopy, based on the nature of the frequency of the electric current. 

#### 3.3.1. Electrical Conductance Measurement

The electrical conductance measurement device uses a single, fixed-frequency alternating current to measure the electrical conductance of a carious tooth [55]. The measuring electrode is designed to fit into deep pits and fissures to be in contact with a minuscule amount of dentinal fluid to complete the circuit [57]. If no carious lesions are present on a tooth and have intact enamel, a circuit of the current flow cannot be completed, making the reading on the device zero. If a lesion is present, a current flow would present and the circuit would be closed, which provides a reading on the device [57]. 

The electrical conductance measurement device allows for the detection of both non-cavitated and cavitated caries lesions on occlusal, proximal, and smooth surfaces. Since the reading correlates to the loss of minerals and increased porosities, electrical conductance measurement enables the assessment of caries severity with a quantitative approach. Moreover, this diagnostic aid can differentiate caries and stains, which visual and fluorescence methods may pick up on [57]. However, there is still much discussion on the cost and feasibility of such technology.

Research on electrical conductance measurement is limited [58]. An in vitro study showed that an electrical conductance measurement device demonstrated a high sensitivity value of 1 and a specificity value of 0.93 in detecting early occlusal caries [57].

#### 3.3.2. Alternating Current Impedance Spectroscopy 

Alternating current impedance spectroscopy measures the impedance or the resistance of the teeth to alternating current. This detection method utilizes multiple frequencies of electricity to produce a spectrum of values, providing more information on the physical and chemical properties of a tooth [55]. A sensing brush can be moved over the suspected carious site, providing a numerical reading supplemented by a colour code that indicates the probability of caries [59]. This method can detect carious lesions at early stages. A systematic review and meta-analysis reported the clinical sensitivity and specificity for detecting occlusal caries to be 0.3–0.92 and 0.75–0.97 in permanent teeth [5]. 

### 3.4. Ultrasound-Based Diagnostic Aids 

Ultrasound has been introduced into dentistry as a diagnostic tool for caries detection by detecting the difference between sound and demineralized hard tissue through the sonic conductivity of longitudinal ultrasonic waves. It utilizes ultrasonic waves which can be transmitted on smooth, flat, or curved hard tissue surfaces. Ultrasound-based diagnostic aids have been developed into a prototype as ultrasonic caries detector. 

Ultrasonic Caries Detector

The ultrasonic probe of the ultrasonic caries detector can be placed at a certain angle to detect caries lesions, particularly approximal carious lesions [60,61]. Ultrasonic caries detectors are relatively simple because the ultrasonic waves are amplified and are significantly greater than that of the background level, allowing for the easier interpretation of wave profiles [60]. Additionally, it is not required for the ultrasonic probe to be placed directly on caries lesions [60], which broadens its clinical application to caries lesions that are difficult to assess. These advantages of adequate directionality, high penetration level, and non-toxicity allow this technology to be a potential caries diagnostic aid [62]. However, ultrasound in the medical field has been reported to have a low spatial resolution, deeming it less competitive [63]. This inability to measure carious lesion depth is another drawback of this digital detection aid [60].

The accuracy of ultrasonic caries detectors was reported in a limited number of studies. Its sensitivity and specificity value for approximal caries were reported to be 0.82 and 0.75, respectively [60].

**Table 1:** Examples of digital diagnostic aids and their energy sources.

Category	Technology	Examples of Commercial Products	Energy Source
**Radiation-based**	Bitewing radiography	Planmeca ProXTM (Planmeca, Helsinki, Finland)	X-ray radiation [18]
	Cone beam computed tomography	Planmeca ProMax 3D (Planmeca, Helsinki, Finland)	X-ray radiation [18]
**Light-based**	Digital transillumination	DIAGNOcam (KaVo Dental GmbH, Birebach/Riẞ, Germany)	780 nm near-infrared light [64]
		i-Tero (Align Technology, San Jose, CA, USA)	850 nm near-infrared light [65]
		VistaCam iX intraoral camera (Durr Dental, Bietigheim-Bissingen, Germany)	850 nm infrared light [66]
	Light-induced fluorescence	Spectra (Air Techniques, New York, NY, USA)	405 nm blue light [67]
		SoproLife (SOPRO, ACTEON Group, La Ciotat, France)	450 nm blue light [37]
		VistaProof (Durr Dental, Bietigheim-Bissingen, Germany)	405 nm blue light [68]
		3Shape TRIOS 4 (3Shape, Copenhagen, Denmark)	415 nm blue light [69]
		Caries Detector (Optica Laser, Sofia, Bulgaria)	390–420 nm near-ultraviolet light [45]
	Laser-induced fluorescence	DIAGNOdent device (KaVo Dental GmbH, Birebach/Riẞ, Germany)	655 nm red-light laser [43]
		DIAGNOdent Pen (KaVo Dental GmbH, Birebach/Riẞ, Germany)	655 nm red-light laser [43]
**Ultrasound-based**	Ultrasonic caries detector	Ultrasonic Caries Detector (Novadent Ltd., Lod, Israel)	Ultrasonic waves [60]
**Electric-based**	Electrical conductance measurement	Ortek ECDTM electronic device (Ortek Therapeutics, New York, NY, USA)	Electric current [60]
	Alternating current impedance spectroscopy	CarieScan PRO^TM^ (CarieScan Ltd., Dundee, Scotland)	Electric current [59]

## 4. Digital Diagnostic Aids under Development

Although several digital diagnostic aids are available, all of them present certain levels of limitation and cannot be applied in all scenarios. Moreover, none of them have presented an ideal performance in detecting dental caries, especially for caries in an early stage. Therefore, many novel digital diagnostic aids for dental caries are under development. The following digital diagnostic aids have the potential for clinical use, but currently no mature products are available. 

### 4.1. Optical Coherence Tomography 

Optical coherence tomography (OCT) produces two or three-dimensional images based on the tissue’s optical absorption and scattering properties [70]. The images are created based on the principle of interferometry, which involves the interaction of the emitting light with backscattered light from a sample to produce light wave interference patterns [71]. The interference patterns are compared with the pattern generated from a reference light to produce a micro-structure profile of biological tissues [72]. 

Swept-source (SS-)OCT can be used for caries detection. It has increased image resolution, speed of imaging, and sensitivity compared with traditional OCT systems [71]. A near-infrared laser is typically used as the light source with a centre wavelength of around 1310 nm [72]. In SS-OCT images of caries detection, demineralized enamel or dentine is presented as a bright zone due to the increased backscatter signal from carious tooth structure [72]. SS-OCT can provide real-time video-rate imaging with an improved overall signal-to-noise ratio of the acquired images, which can be beneficial to clinical applications [72]. An in vitro study showed that SS-OCT showed a higher sensitivity than visual inspection for caries at all severity levels [72]. When compared with bitewing radiography, an in vivo study confirmed that SS-OCT is more reliable and accurate when detecting proximal caries [71]. Although current in vivo research for OCT is scarce, advancement in OCT systems paves the way for more non-invasive digital caries detection aids. However, this technology has not yet reached the stage where it is suitable as a commercial clinical caries detection aid [73].

### 4.2. Laser-Related Caries Detection

Apart from the laser-induced fluorescence, lasers can be used to induce other signals for caries detection. A laser can induce thermal signals, acoustic signals, photonic signals, etc., on the tooth surface to facilitate caries detection. 

#### 4.2.1. Laser-Induced Thermal Imaging 

Thermal imaging technology is based on the principle that the degree of porosities in a carious lesion affects the amount of water stored in a tooth and, therefore, its temporal profile [74]. The temporal profile correlates with the continuous evaporation of water from the porosities of dental tissues, leading to thermodynamic changes on the tooth surface until a new equilibrium is established when the tooth is dry [74]. Thermal imaging technology works by either capturing the temporal profile of the chronological evaporation of a carious tooth surface as it dries or by sensing the tooth’s temporal profile immediately following exposure to a heat pulse [74]. 

#### 4.2.2. Frequency-Domain Laser Infrared Photothermal Radiometry and Modulated Luminescence Technology 

Photothermal radiation (PTR) utilizes modulated thermal infrared response, also known as the black body or Plank radiation, which results from a specimen that is repeatedly irradiated. The emitting black body or Plank radiation is the thermal electromagnetic radiation within or surrounding that specimen when it is in thermodynamic equilibrium with its environment. It has a specific constant and intensity that depends solely on the temperature of the specimen. When the specimen absorbs radiation energy, it is converted into thermal energy which can be observed as a change in temperature of the specimen surface. This energy conversion can be measured by an infrared detector using the PTR signal [75]. Recent caries detection technology combines PTR with modulated luminescence technology (LUM), which measures the wavelength emitted when the absorbed optical energy from a laser source is converted to radiation energy. This can be detected using a photodetector with a LUM signal [75].

Frequency-domain laser infrared photothermal radiometry and modulated luminescence technology (FD-PTR/LUM) can detect pit and fissure caries up to 5 mm below a tooth surface. According to an in vitro study, this technology can effectively differentiate between sound teeth surfaces or lesions on the outer half of enamel and lesions extending to the middle of the enamel or deeper [48]. Studies also demonstrated higher sensitivity and specificity values for FD-PTR/LUM in detecting early occlusal caries compared with visual examination, radiography, and laser fluorescence methods [76]. For approximal caries detection, the sensitivity value for FD-PTR/LUM is higher than visual examination and radiography, while the specificity value is similar to radiography but significantly higher than visual inspection [76]. However, the fluctuations in temperature and humidity of the oral cavity can influence the temperature readings of FD-PTR/LUM [48].

#### 4.2.3. Laser-Induced Breakdown Spectroscopy

Caries detection can be achieved via laser-induced breakdown spectroscopy (LIBS) with neodymium-doped yttrium aluminium garnet (Nd: YAG) laser. This approach analyses the spectral changes of element contents in a tooth sample, typically in enamel. Enamel consists of matrix elements such as calcium and phosphorus in the form of hydroxyapatite and non-matrix elements, such as potassium, magnesium, zinc, and carbon. [77]. Each element in the tooth has its own specific absorbed wavelength. The change in the relative concentration of the elements can indicate whether the tooth is carious or healthy. For example, a decrease in matrix elements and an increase in non-matrix elements indicates that the sample is carious [77]. Such technology would enable the dentist to monitor the change in tooth structure during caries and plaque removal in real-time and in vivo [77]. 

#### 4.2.4. Laser-Induced Acoustic Spectroscopy

Laser-induced acoustic spectroscopy can evaluate dental hard tissue’s properties and thickness for the diagnosis of dental caries. When a tooth is irradiated with a pulse laser, the laser energy would be absorbed leading to an increase in localized temperature and thermal expansion, causing the excitation of acoustic waves [62]. A decayed tooth exhibits alternated time and frequency domains of acoustic waves. A Rayleigh wave is a type of surface acoustic wave that is propagated on dental tissues when irradiated by a laser [61]. The velocity field of the Rayleigh waves can indicate the demineralization degree, depth, and morphology of a carious lesion [62]. Both carbon dioxide pulse laser and Nd: YAG laser have been used in this technology to excite acoustic waves of a decayed tooth. This diagnostic method primarily identifies early carious lesions, where only the mineral content has changed. An in vitro study examined the presence of incipient and advanced caries using a laser beam light source of selective wavelength within the infrared and visible light spectrum [61,78]. The main advantage of acoustic imaging is its capability for higher penetration depth and better spatial resolution [79]. More studies are needed for the development of photoacoustic imaging into an accessible clinical tool for operators. 

### 4.3. Diagnostic Interpretation Aids

Novel strategies that enhance the accuracy in the interpretation of the results of digital diagnostic aids have also been developed. These diagnostic interpretation aids were applied based on the caries-detecting results of the digital diagnostic aids. For example, automatic exposure compensation (AEC) is an ancillary digital tool to improve image quality and the accuracy of diagnosis for digital intraoral radiographs [80,81]. Artificial intelligence was employed to improve the accuracy, consistency, and efficiency of digital diagnostic aids by reducing the potential errors of human assessment [82]. 

## 5. Limitations of This Review

While this review provides a comprehensive overview of the various digital diagnostic aids clinically available for the detection of dental caries, its limitations should be addressed.

Many clinically available digital caries diagnostic aids are recently developed and there is still limited clinical data with regards to their diagnostic accuracy and the potential impact on clinical application such as patient experience and satisfaction. The financial and practical implications of adopting these diagnostic aids were not fully discussed as well. It is very much anticipated that further research can be conducted to develop and test efficient and effective caries diagnosis in the future.

This review focuses on the introduction of digital diagnostic aids and their clinical application. It should be noted that some strategies that enhanced the accuracy in the interpretation of the results of the digital diagnostic aids has also been developed. For example, automatic exposure compensation (AEC) is an ancillary digital tool to improve image quality and the accuracy of diagnosis for digital intraoral radiographs [80,81]. Artificial intelligence was used improve the accuracy, consistency, and efficiency of the digital diagnostic aids by reducing the potential errors of human assessment [82]. These strategies were not under the scope of the present review.

## 6. Summary

Digital diagnostic aids for dental caries detection have been evolving. Radiation-based aids, light-based aids, ultrasound-based aids, and electric-based aids are now available as digital diagnostic aids for dental caries detection. These available digital diagnostic aids present advantages and certain levels of limitation. They facilitate caries detection for clinicians by acting as supplementary approaches to conventional caries detection. However, none of them have presented an ideal performance in detecting dental caries, especially for caries in an early stage. Therefore, various novel digital diagnostic aids for dental caries are under development and show potential for clinical application. Furthermore, this review aims to allow clinicians a more comprehensive review of currently available digital diagnostic aids, and to acknowledge and compare various listed novel technologies to facilitate easier early caries detection, and the subsequent prevention of dental caries.

## Data Availability

Not applicable.
